# Winning and losing: differences in reward and punishment sensitivity between smokers and nonsmokers

**DOI:** 10.1002/brb3.285

**Published:** 2014-09-19

**Authors:** Laura E Martin, Lisa S Cox, William M Brooks, Cary R Savage

**Affiliations:** 1 Hoglund Brain Imaging Center, University of Kansas Medical CenterKansas City, Kansas; 2 Department of Preventive Medicine and Public Health, University of Kansas Medical CenterKansas City, Kansas; 3 Department of Neurology, University of Kansas Medical CenterKansas City, Kansas; 4 Center for Health Behavior Neuroscience, University of Kansas Medical CenterKansas City, Kansas; 5 Department of Psychiatry and Behavioral Sciences, University of Kansas Medical CenterKansas City, Kansas

**Keywords:** fMRI, punishment sensitivity, reward sensitivity, smoking

## Abstract

**Background:**

Smokers show increased brain activation in reward processing regions in response to smoking-related cues, yet few studies have examined secondary rewards not associated with smoking (i.e., money). Inconsistencies exist in the studies that do examine secondary rewards with some studies showing increased brain activation in reward processing brain regions, while others show decreased activation or no difference in activation between smokers and nonsmokers.

**Aims:**

The goal of the current study is to see if smokers process the evaluation and delivery of equally salient real world rewards similarly or differently than nonsmokers.

**Methods:**

The current study employed functional magnetic resonance imaging (fMRI) to examine brain responses in smokers and nonsmokers during the evaluation and delivery of monetary gains and losses.

**Results:**

In comparison to nonsmokers, smokers showed increased activation in the ventromedial prefrontal cortex to the evaluation of anticipated monetary losses and the brain response. Moreover, smokers compared to nonsmokers showed decreased activation in the inferior frontal gyrus to the delivery of expected monetary gains. Brain activations to both the evaluation of anticipated monetary losses and the delivery of expected monetary gains correlated with increased self-reported smoking craving to relieve negative withdrawal symptoms and craving related to positive aspects of smoking, respectively.

**Discussion:**

Together these results indicate that smokers are hyperresponsive to the evaluation of anticipated punishment and hyporesponsive to the delivery of expected rewards. Although further research is needed, this hypersensitivity to punishments coupled with increased craving may negatively impact quit attempts as smokers anticipate the negative withdrawal symptoms associated with quitting.

## Introduction

Individual differences in reward and punishment sensitivity influence how and why individuals make decisions. Given that a substantial proportion of the population continues to smoke despite known risks, examining individual differences in reward and punishment sensitivity between smokers and nonsmokers may provide insight into why some individuals continue to smoke while others never start smoking.

Studies of reward processing consistently demonstrate that the neural systems of motivation respond to reward anticipation as well as reward delivery (Schultz et al. 1997; Knutson et al. 2001). Anticipation in nicotine addiction can be seen in studies of neural responses to smoking-related cues in which presentations of smoking images evoke the pleasure that is anticipated with future smoking. Studies examining brain responses to smoking cues in smokers show that motivation regions respond differently based on smokers’ expectations to smoke during an experiment (Wilson et al. 2005; McBride et al. 2006), motivation to quit smoking (Wilson et al. 2012b), self-report levels of nicotine dependence (Smolka et al. 2006; McClernon et al. 2008; Goudriaan et al. 2013), and smoking ambivalence (Wilson et al. 2012a). A recent meta-analysis of fMRI smoking cue-reactivity studies verified that smoking cues reliably activate brain regions related to reward processing (anterior cingulate cortex and medial prefrontal cortex), memory (parahippocampal gyrus), control (dorsolateral prefrontal cortex), and interoceptive awareness (insula, dorsal striatum) (Engelmann et al. 2012).

Models of addiction posit that addiction is associated with increased sensitivity to the anticipation of drug reward and decreased sensitivity to other rewards (e.g., food, sex, etc.) (Baler and Volkow 2006). Despite connections between reward processing and addiction, few studies have directly examined differences in function of the neural systems of reward to monetary gains or losses among cigarette smokers. Given that monetary rewards are salient to both smokers and nonsmokers, whereas smoking cues are salient only to smokers, monetary rewards provide a real world framework to directly compare reward-related brain activations between smokers and nonsmokers. Monetary rewards have been used to study reward processing in other addictions, such as gambling and alcohol. Behavioral studies using monetary rewards show that smokers compared to nonsmokers discount the value of delayed rewards and choose immediate rewards more frequently than delayed rewards (Bickel et al. 1999; Mitchell 1999; Field et al. 2006). However, neuroimaging studies of monetary reward processing in smokers show inconsistencies. Some studies show increased (Luijten et al. 2013; Rose et al. 2013), some show decreased (Wilson et al. 2008; Buhler et al. 2010; Luo et al. 2011; Addicott et al. 2012; Lessov-Schlaggar et al. 2013), and others show no change (Peters et al. 2011) in brain activation to monetary gains. Similar inconsistencies are present for studies examining monetary losses (Lessov-Schlaggar et al. 2013; Luijten et al. 2013; Rose et al. 2013).

The goal of this study is to see if smokers process the anticipation and delivery of equally salient real world rewards similarly or differently than nonsmokers. The study design allows separation of cue evaluation (e.g., anticipation) and receipt of nonsmoking rewards and punishments. If smokers show increased activation to the cue evaluation and delivery of rewards, results support an overall drive to attain rewards regardless of consequences. In contrast, if smokers show increased activation to the cue evaluation and delivery of punishments, results support an overall drive to avoid punishment. On the other hand, if smokers show decreased activation to the cue evaluation and delivery of rewards and/or punishments, results support an overall dampening of motivational responses to acquire rewards and avoid punishments. By examining the cue evaluation and delivery of monetary rewards and punishments, the current study extends previous research by examining the interaction between expectation and valence of motivating real world stimuli between smokers and nonsmokers. In addition, the current study examined the association between craving and brain responses to the cue evaluation and delivery of nondrug rewards. This approach will be a first step toward understanding the role craving may play in reward and punishment sensitivity among smokers.

## Methods

### Participants

The University of Kansas Medical Center Human Subjects Committee approved the current study. Informed consent was obtained for all participants. We enrolled 20 smokers (10 female) reporting smoking at least 10 cigarettes per day (CPD) for at least 6 months and 19 nonsmokers (nine female) who reported smoking less than 100 cigarettes in their lifetime with no smoking in the past 6 months. All participants were right-handed. Exclusion criteria for both groups included: self-reported serious medical illness unsuitable for the MRI scanner based on best clinical judgment, any neurologic or psychiatric disorder, diabetes, known heart disease, high blood pressure, any thyroid condition, significant visual impairment, seizure disorder, current psychotropic or cardiovascular medication use, and current alcohol or other substance abuse. One smoker and two nonsmokers did not complete the MRI portion of the study due to claustrophobia. In addition, one smoker was excluded from data analysis due to technical problems with the stimulus presentation and two smokers were excluded from data analysis due to excessive movement (greater than 3 mm) during the scan. The current analyses included the remaining 16 smokers (mean CPD = 15.17; SD = 4.91) and 17 nonsmokers.

### Procedures

Smokers and nonsmokers completed the same procedures. During the first 2 h, participants completed the Vocabulary and Matrix Reasoning sections of the WAIS-III and questionnaires followed by an hour of MRI testing. All participants were compensated $50 for their time commitment and had the opportunity to increase the amount earned by up to $25 based on their performance during the modified Reward Prediction Task (RPT) (Martin and Potts 2004, 2011; Potts et al. 2006, 2010; Martin et al. 2009). Smokers were allowed to smoke immediately before the testing began and not again until they completed the study about 3.5 h later. In addition, smokers completed questionnaires assessing dependence and craving. Smoking dependence was measured using the Fagerstrom Test for Dependence (FTND) (Heatherton et al. 1991). Craving was measured using the Brief Questionnaire of Smoking Urges (QSU-Brief) (Cox et al. 2001) at the beginning of the study appointment, immediately before the MRI, and immediately after the MRI. The QSU-Brief contains two factors. Factor 1 assesses craving associated with positive reinforcement of smoking and Factor 2 assesses craving associated with relief of negative affect resulting from smoking.

### fMRI reward prediction task

**Figure 1:**
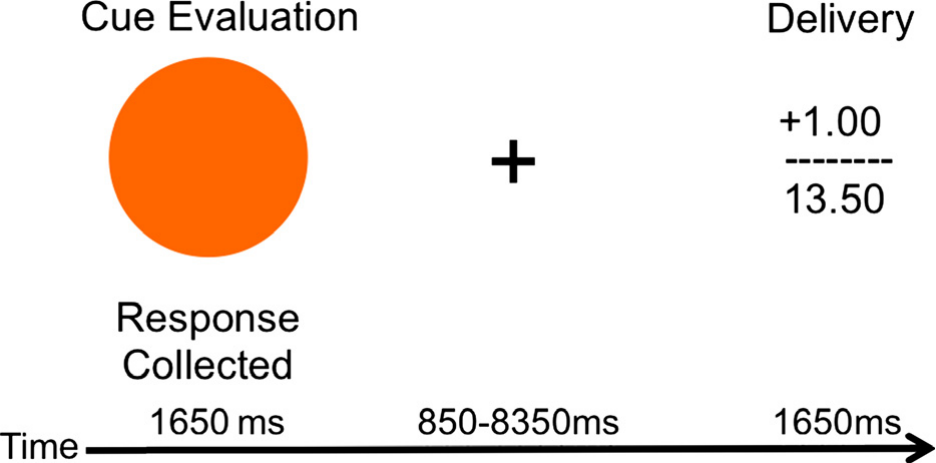
Reward prediction task in which a colored circle predicted the delivery of a monetary gain or loss followed by a brief anticipation period and feedback indicating the amount earned on the current trial and the total for the current block of trials. The expected outcome was delivered on 75% of the trials.

The RPT is based on Martin and Potts (2004, 2011), Potts et al. (2006, 2010), and Martin et al. (2009). Participants were presented with cues (blue and orange circles) that correctly predicted the delivery of a monetary gains or losses with 75% accuracy (e.g., predicted gains and losses). The remaining 25% of the trials resulted in the delivery of unexpected monetary gains (e.g., expecting to win and actually lost) and unexpected monetary losses (e.g., expecting to lose and actually won). Prior to entering the scanner, participants were told which cues predicted monetary gains and which predicted losses and that some trials would result in unexpected outcomes. In addition, participants completed 16 practice trials to make sure they understood the task instructions and that the effects measured during scanning were related to gains and losses as opposed to learning effects. The predictor was presented for 1650 msec during which the participant indicated with a keypress whether the cue predicted a gain or a loss. This was followed by a fixation cross for 850–8350 msec (average cue evaluation duration = 3350 msec), which served as the cue evaluation phase of the trial. The participant then received feedback for 1650 msec indicating how much he/she won or lost on the current trial and his/her total for the current block of trials (Fig.1). Participants had no control over whether they won or lost. The color of the circle and keypress required for gains and losses were counterbalanced across participants. Delays ranging from 0 to 13 sec (average intertrial interval duration = 2865 msec) were inserted between trials to jitter trial presentation. The optimal stimulus and delay timing was determined using analysis of functional neuroimage (AFNI) stimulus timing program RSFgen.

Participants began each fMRI run with $12.50 in their bankroll at the beginning of the task and received $1 gains and losses during the task. Incorrect responses resulted in $0.25 rewards and $1.75 punishments. In addition, incentives were given for fast responses (≤500 msec) with larger gains ($1.50) and smaller losses ($0.50). Incentives were used to keep participants engaged in the task. The percentage of trials where participants earned bonus incentives earned did not differ between groups (smokers: mean = 51%, SD = 17%; nonsmokers: mean = 58%, SD = 28%, *P *=* *0.36). Therefore, results on these trials were not analyzed separately. Bankroll totals were reset at the beginning of each of the four fMRI runs. Each run consisted of 30 trials and was about 5-min long. At the end of the experiment participants were paid what they earned on a randomly selected fMRI run.

### fMRI data acquisition and analysis

Scanning was performed at the University of Kansas Medical Center's Hoglund Brain Imaging Center on a 3-Tesla head-only Siemens Allegra scanner (Siemens, Erlangen, Germany) fitted with a quadrature head coil. T1-weighted anatomic images (3D MPRAGE, TR/TE = 23/4 msec, flip angle = 8°, FOV = 256 mm, matrix = 256 × 192, slice thickness = 1 mm) were used for slice localization for the functional scans, Talairach transformation, and coregistration with fMRI data. Following structural scans, gradient echo blood-oxygen-level-dependent (BOLD) scans were acquired in 40 contiguous slices at a 40° angle to the AC-PC line (repetition time/echo time [TR/TE] = 2000/40 msec, flip angle = 90°, field of view [FOV] = 220 mm, matrix = 64 × 64, slice thickness = 3 mm, 0.5 skip, in-plane resolution = 3.75 × 3.75 mm, spatial filter = 1.0 HZ). All functional scans were acquired at a 40° angle to the AC-PC line to minimize susceptibility artifact in orbitofrontal cortex. Based on recommendations by Deichmann et al. (2003), all participants were positioned in the scanner so that the angle of the AC-PC plane was between 17 and 22° in scanner coordinate space. This angle was verified with a localization scan.

Preprocessing and statistical analyses were performed in AFNI. Preprocessing steps included slice time correct-ion, motion correction, and spatial normalization. Spatial normalization was done by transforming participants‘ anatomical scans to Talairach stereotaxic space using AFNI's automated algorithm (@auto_tlrc) and this transformation was applied to the participants‘ functional scans. Statistical contrasts were conducted using multiple regression analysis with motion parameters included as nuisance regressors. Regressors representing the experimental conditions for both the cue evaluation and delivery phase of each trial were modeled with a hemodynamic response filter and entered into the multiple-regression analysis using a random-effects model. Duration modulation regression in AFNI was used so that the cue evaluation phase included the time from the presentation of the cue until the participant responded. The cue evaluation phase included two experimental conditions: gains and losses. The cue evaluation phase only include the time until the participant responded to avoid collinearity issues between the cue evaluation phase and the delivery phase of each trail. The delivery phase included four experimental conditions: expected gains, expected losses, unexpected gains, and unexpected losses. Incorrect trials were not included in the analysis due to the small number of trials across conditions.

Analyses focused on group differences in the cue evaluation phase and delivery of gains and losses phase of the task, as well as the difference between gains and losses. In addition, analyses examined differences in brain response (i.e., percent signal change) when outcomes were better than expected (unexpected gains – expected losses) and when outcomes were worse than expected (unexpected losses – expected gains). Within group analyses are also provided. Analysis focused on a priori reward processing regions, including medial and lateral prefrontal cortex, anterior cingulate, and limbic regions using small volume corrections for multiple comparisons. Masks were created using AFNI's *whereami* function and the TT_Daeman atlas locations. Specifically, a mask of prefrontal and limbic regions including the medial frontal gyrus, middle frontal gyrus, superior frontal gyrus, subcallosal gyrus, anterior cingulate cortex, ventral striatum, caudate, and putamen was created and multiple comparisons were corrected within this mask (*P*_corrected_* *< 0.05, *P*_voxelwise_ < 0.005). Activations were corrected for multiple comparisons within the masks based on Monte Carlo simulations using AFNI's 3dClustSim including smoothness estimates from the functional scan residuals.

In addition, an ROI analysis was performed for the nucleus accumbens using AFNI's *whereami* to extract percent signal change from the left and right nucleus accumbens. These values were then imported into SPSS to examine within- and between-group activations during the cue evaluation of monetary gains and losses as well as the delivery of monetary gains and losses.

### Correlations with smoking measures

Follow-up analyses were conducted to examine associations between brain responses and self-reported change in craving measured by the QSU-Brief Factor 1 and 2 scores. Craving change was measured by subtracting QSU-Brief scores at the beginning of the testing session from scores measured following the scan. Mean percent signal change values were extracted for each individual from functionally defined regions of interest in reward processing regions showing significant group differences as well as the left and right nucleus accumbens. Correlation analyses were performed using SPSS 20.0 for Macintosh (Statistical Package for the Social Sciences, Chicago, IL).

## Results

### Demographics

Groups were not significantly different in terms of age (smokers: mean = 31.4, SD = 9.82, nonsmokers: mean = 33.73, SD = 10.29; *P *=* *0.47) and Wechsler Intelligence Scale-III (WAIS III) vocabulary scores (smokers: mean = 10.9, SD = 3.13; nonsmokers: mean = 12.52, SD = 3.13, *P = *0.12) and matrix reasoning scores (smokers: mean = 12.11, SD = 3.33; nonsmokers: mean = 12.11, SD = 3.01, *P *=* *0.75).

### Nicotine dependence and craving

FTND scores indicated that the smokers had relatively low levels of nicotine dependence (mean = 3.56, SD = 1.90) (Fagerstrom et al. 1990). Results of the QSU-Brief demonstrated that craving related to positive reinforcement of smoking increased from the start of the study appointment to the end of the study appointment approximately 3.5 h later (*F*(1, 15) = 23.72, *P* < 0.001). Specifically craving related to the positive reinforcement of smoking increased significantly (*P* < 0.05) from the beginning of the appointment (mean = 22, SD = 5.58) to immediately before the scan (mean = 52.56, SD = 8.1) and from immediately before the scan to the end of the appointment (mean = 65.25, SD = 7.89). Craving related to relief of negative affect and withdrawal increased significantly from the start of the study appointment to the end of the study appointment (*F*(1, 15) = 5.523; *P* < 0.01). Craving related to the relief of negative affect did not significantly change (*P* = 0.20) from the beginning of the appointment (mean = 12.38, SD = 19.20) to immediately before the scan (mean = 17.81, SD = 19.89). However, craving did significantly increase (*P* < 0.05) immediately before the scan to the end of the appointment (mean = 25.06, SD = 26.54).

### Behavioral results

No significant differences were found between smokers and nonsmokers for accuracy (*P *=* *0.14) or reaction time (*P *=* *0.26). On average, participants were 95% accurate (range = 70–100%) and had an average reaction time of 492 msec (range = 396–648 msec). Accuracy did not differ between anticipated gain and anticipated loss (*P *=* *0.50), and no interaction was found between anticipated outcome and group (*P *=* *0.20). Accuracy did not change between runs (*P *=* *0.40). Overall participants showed a trend (*P* = 0.08) toward faster reaction times when anticipating rewards (mean = 486 msec, SD = 10 msec) compared to punishments (mean = 498 msec, SD = 9 msec). However, no significant interaction effects in reaction times were found between anticipated outcomes and group (*P *=* *0.19). On average participants earned $19.95 (range $10.50–$27). Participant earnings did not differ between smokers and nonsmokers (*P *=* *0.14).

### Cue evaluation

#### Smokers

No regions were found to show significant differences between the cue evaluation of anticipated monetary gains compared to losses among smokers (Table 1). Region of interest analyses in the nucleus accumbens showed significant changes from baseline during the cue evaluation of anticipated losses in the left (*t*(15) = −3.06, *P *<* *0.01) and right accumbens (*t*(15) = −2.97, *P *<* *0.01), but not to the cue evaluation of anticipated gains. In addition, the left nucleus accumbens showed greater deactivation to anticipated losses compared to gains (*t*(15) = 2.75, *P* < 0.05).

**Table 1:** Smokers gains versus losses.

Region	*x*	*y*	*z*	Number of voxels	*z*-score
Anticipation gains vs. losses					
	No significant differences				
Delivery expected gain–loss					
Middle frontal gyrus	30	31	45	34	3.76
Superior frontal gyrus	−19	52	10	30	4.67
Anterior cingulate cortex	−5	38	3	23	3.39
Putamen	−19	10	3	32	4.04
	23	10	10	29	4.20
Caudate	12	10	10	8	4.26
Delivery unexpected gain–loss					
	No significant differences				
Delivery better than expected					
	No significant differences				
Delivery worse than expected					
Putamen	16	6	−4	86	−5.17
	−16	3	10	78	−4.88

#### Nonsmokers

**Figure 2:**
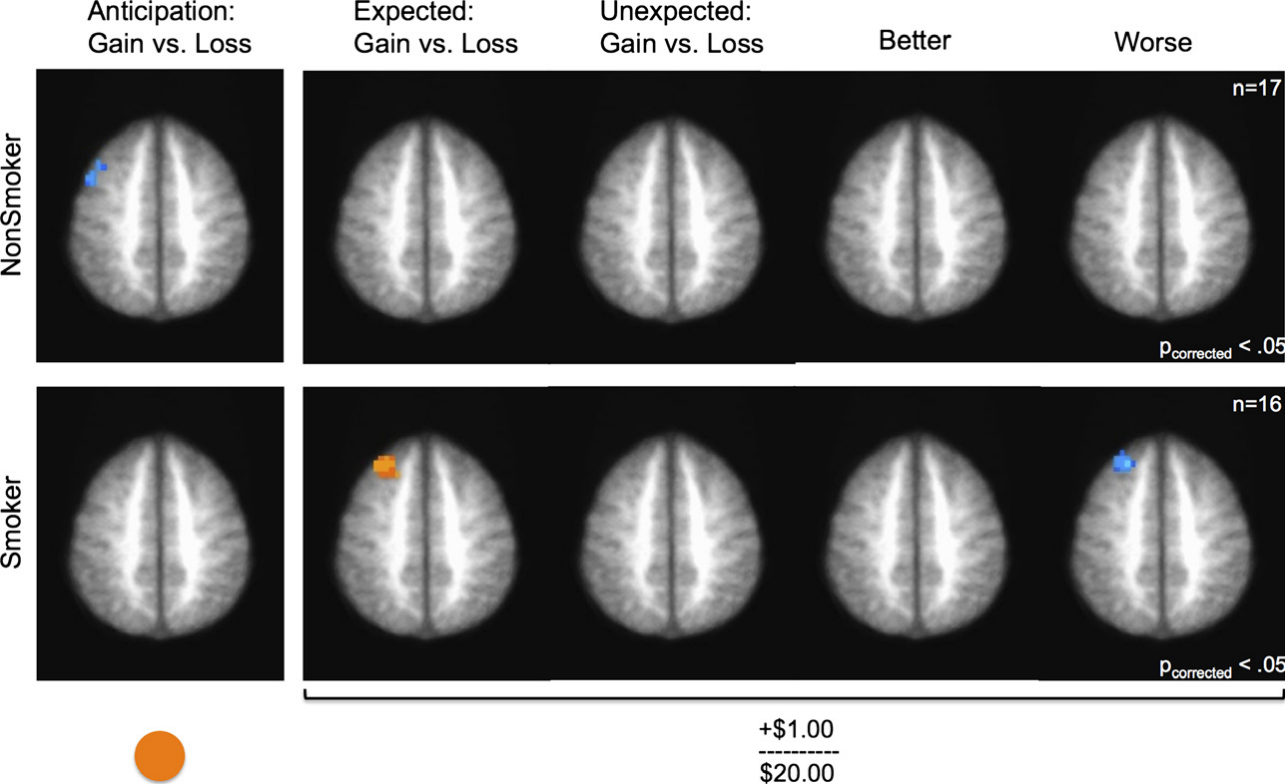
Lateral prefrontal activations during the anticipation and delivery of monetary gains and losses in smokers and nonsmokers.

Among nonsmokers, greater activation (i.e., percent signal change) was found in the right middle frontal gyrus to the cue evaluation of anticipated monetary losses compared to gains (Table 2; Fig.2). No significant changes from baseline were found in the nucleus accumbens to the anticipation of gains or losses.

**Table 2:** Nonsmokers gains versus losses

Region	*x*	*y*	*z*	# of Voxels	*z*-score
Anticipation gain–loss					
Middle frontal gyrus	37	17	48	22	−4.48
Delivery expected gain–loss					
Putamen	−16	13	−4	7	3.44
Caudate	12	13	3	12	3.65
Delivery unexpected gain–loss					
Putamen	−19	13	3	20	3.60
Delivery better than expected					
	No significant differences				
Delivery worse than expected					
Putamen	−16	10	3	50	−3.80
	−26	−4	−4	7	−3.27
	16	10	−1	30	−3.64
Caudate	9	13	3	22	−3.84

#### Smokers versus Nonsmokers

**Figure 3:**
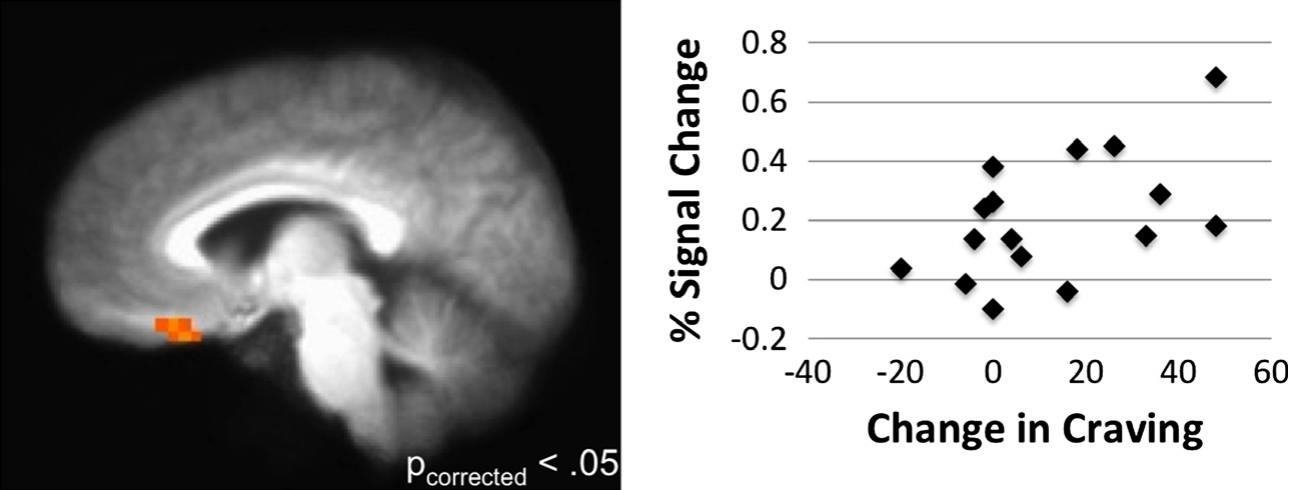
Ventromedial prefrontal activation showing greater response to the anticipation of monetary losses in smokers compared to nonsmokers and a positive correlation with change in craving related to relieving negative withdrawal symptoms (QSU-Brief, Factor 2).

Smokers compared to nonsmokers showed greater activation during the cue evaluation of anticipated monetary losses in ventromedial prefrontal cortex (vmPFC)/Brodmann area 25; *x*, *y*, *z* = −2, 24, −18; voxels = 20; *z* = 4.11; Fig.3). Smokers and nonsmokers did not differ in response to the cue evaluation of anticipated monetary gains compared to losses. Furthermore, no significant differences in activation of prefrontal or limbic regions were found between smokers and nonsmokers during the cue evaluation of anticipated monetary gains. Region of interest analyses were conducted in the nucleus accumbens and showed greater deactivation to the cue evaluation of anticipated losses in smokers than nonsmokers in the right nucleus accumbens (*t*(31) = 2.28, *P *<* *0.05). In addition, the right nucleus accumbens in smokers showed a greater deactivation to the evaluation of monetary losses compared to gains, whereas nonsmokers showed no difference in response to cue evaluation of anticipated gains or losses (*t*(31) = −2.081, *P *<* *0.05) with smokers showing greater deactivation to losses compared to gains and nonsmokers showing no difference in response to losses compared to gains.

### Delivery

#### Smokers

**Figure 4:**
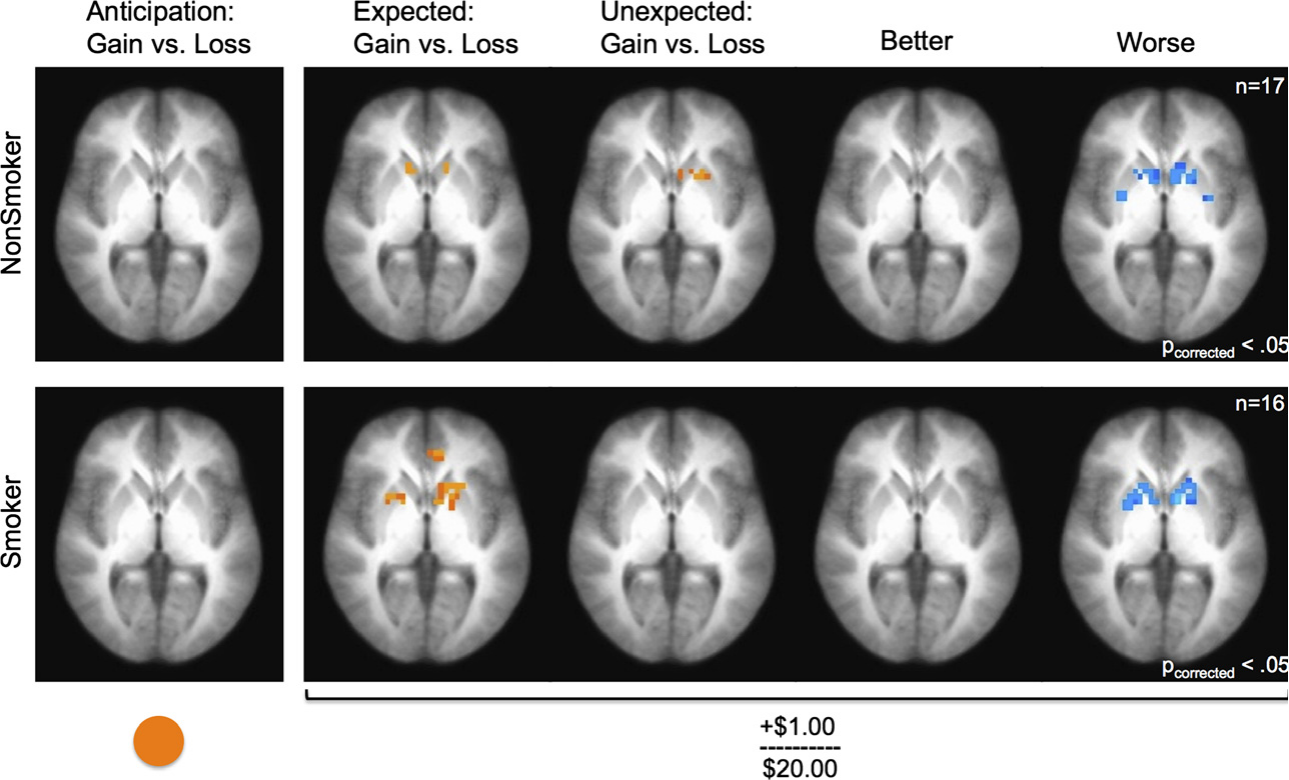
Limbic activations during the anticipation and delivery of monetary gains and losses in smokers and nonsmokers.

Smokers showed increased activation during the delivery of expected monetary gains compared to losses in prefrontal and limbic regions including the MPFC, ACC, superior frontal gyrus, putamen, and caudate (Figs.2, 4). No significant differences were found during the delivery of unexpected monetary gains compared to losses or when outcomes were better than expected (i.e., expected to lose and then won). On the other hand, when outcomes were worse than expected (i.e., expected to win and then lost), smokers showed decreased activation in the superior frontal gyrus and putamen (Figs.2, 4). Table 1 summarizes brain regions demonstrating differences between monetary gains and losses among smokers. In addition, smokers showed significant changes from baseline in the left nucleus accumbens during the delivery of unexpected punishments (*t*(15) = −3.48, *P *<* *0.01). No significant differences were found during the delivery of unexpected rewards.

#### Nonsmokers

During the delivery of expected monetary gains and losses, nonsmokers showed increased activation to gains compared to losses in the caudate. Moreover, nonsmokers showed increased activation to the delivery of unexpected gains compared to losses in the putamen. When outcomes were better than expected (i.e., expected loss and then gained), nonsmokers showed increased activation in the left superior frontal gyrus. When outcomes were worse than expected (i.e., expected gain and then lost), nonsmokers showed decreased activation in the putamen and caudate (Fig.4). Table 2 summarizes brain regions demonstrating differences between monetary gains and losses for nonsmokers. Furthermore, nonsmokers showed deactivation in the left nucleus accumbens to the delivery of expected losses compared to baseline and greater deactivation to the delivery of expected losses compared to gains, yet showed no significant differences in response to unexpected outcomes.

#### Smokers versus Nonsmokers

During the delivery of expected monetary gains, smokers compared to nonsmokers showed less activation in the inferior frontal gyrus/Brodmann areas 45 and 47 (*x*, *y*, *z* = 51, 20, −1; voxels = 21; *z* = −3.38). No significant group differences were found when comparing activation during the delivery of monetary gains to monetary losses. Furthermore, no significant group differences were found during the delivery of monetary losses. Region of interest analyses were conducted in the nucleus accumbens and showed greater deactivation in the left nucleus accumbens to the delivery of unexpected punishment in smokers compared to nonsmokers (*t*(31) = 2.570, *P *<* *0.05). No significant differences were found in response to unexpected gains or expected gains or losses.

### Correlations with measures of smoking craving

Among smokers, correlations examined associations between changes in measures of craving (i.e., QSU-Brief) and brain responses (i.e., average percent signal change for the vmPFC) in regions that showed differences between smokers and nonsmokers during cue evaluation and delivery of monetary gains and losses. Significant correlations were found between changes in craving related to smoking to relieve negative affect (QSU-Brief, Factor 2) and brain responses to the cue evaluation of the anticipation of monetary losses in vmPFC (*r* = 0.53, *P* < 0.05; Fig.2). Specifically, smokers showing the greatest increase in craving also showed the largest activations to the cue evaluation of anticipated monetary losses. In addition, significant correlations were found between changes in craving associated with the positive reinforcement of smoking (QSU-Brief, Factor 1) and the delivery of expected monetary gains in the inferior frontal gyrus (*r* = 0.551, *P* < 0.05; Fig.3). No significant correlations were found between changes in craving and nucleus accumbens response to the cue evaluation or delivery of monetary losses or the delivery of unexpected losses.

## Discussion

This study examined whether smokers and nonsmokers process the anticipation and delivery of real world rewards (i.e., money) in the same way or differently. The main finding of our study was that smokers compared to nonsmokers showed greater activations in the vmPFC, a region related to evaluation of motivational stimuli, during the cue evaluation of monetary losses. Moreover, increased activation during the cue evaluation of monetary losses was associated with increased craving to relieve negative affect associated with short-term smoking abstinence (about 2 h). These results extend previous findings from cue-reactivity studies to nondrug cues, showing that context, in this case craving state, influences brain responses to the cue evaluation of anticipated monetary losses. In contrast, smokers showed less activation compared to nonsmokers when expected monetary gains were delivered in the inferior frontal gyrus, a region associated reward evaluation, during reward delivery. Increased activation in the inferior frontal gyrus to the delivery of expected rewards was associated with increased craving related to positive reinforcement of smoking. Our results are similar to previous studies in smokers with low levels of dependence and reduced prefrontal response to the delivery of monetary gains (Buhler et al. 2010).

In addition, within group analyses revealed that both smokers and nonsmokers showed increased activation in the caudate to the delivery of monetary gains compared to losses, as well as decreased activation in the putamen when outcomes were worse than expected. However, no regions were found within the smokers that responded differentially to outcomes that were better than expected. Moreover, the nucleus accumbens was found to respond preferentially to the cue evaluation of anticipated losses as well as the delivery of unexpected losses in smokers compared to nonsmokers. Together these results further support a hypersensitivity to punishments among smokers.

The current study extends previous research that focus only on monetary gains (Buhler et al. 2010; Luo et al. 2011; Peters et al. 2011; Addicott et al. 2012) to examine brain activation to the cue evaluation (e.g., anticipating) and delivery of monetary losses. Existing results in smokers are inconsistent with some studies showing increased (Luijten et al. 2013; Rose et al. 2013), some showing decreased (Addicott et al. 2012; Buhler et al. 2010; Lessov-Schlaggar et al. 2013; Luo et al. 2011; Wilson et al. 2008), and others showing no change (Peters et al. 2011) in brain activation to the anticipation and delivery of monetary gains. Inconsistencies in brain responses to monetary gains and losses are likely driven by differences in study design and smoking behaviors of participants. For instance, studies showing decreased brain activation to monetary gains among smokers have used tasks where smokers make a decision such as guessing the value of card (Lessov-Schlaggar et al. 2013; Wilson et al. 2008), or making a decision between a “safe” (e.g., 30% probability of winning big) versus a “risky” (e.g., 10% probability of winning a large reward) decisions (Addicott et al. 2012). On the other hand, studies showing increased activation to the anticipation of monetary gains included both reward and neutral trials (Rose et al. 2013). In terms of smoking behaviors, studies vary based on the inclusion of occasional smokers (i.e., smoked fewer than six cigarettes/week) (Buhler et al. 2010), abstinent smokers (Addicott et al. 2012), and administration of nicotine patch during the scanning session (Rose et al. 2013).

Results of the current study demonstrate that smokers who smoked at least 10 cigarettes per day, show low-to-moderate levels of dependence according the FTND, were scanned approximately 2 h after their last cigarette, and expect to smoke within an hour of completing the imaging show increased sensitivity (indexed by brain activation) to the cue evaluation of punishment and decreased sensitivity to the delivery of monetary rewards. Although the current study was not designed to test the influence of experimental design on brain activations, the results indicate that craving can influence brain responses to monetary rewards by increasing sensitivity to the cue evaluation of monetary losses and the delivery of monetary rewards in reward processing brain regions which has not been previously demonstrated.

Limitations of the current study included the absence of a neutral condition (no gain/no loss) that would have provided a more meaningful contrast than baseline fixation, particularly considering that many of the same areas that respond to monetary gains also respond to monetary losses (e.g., prefrontal and limbic regions). However, in the context of winning and losing money a truly neutral stimulus is difficult because a no gain/no loss condition is a punishment in the context of anticipated winning but a reward in the context of losing. The absence of significant differences in classic reward processing regions such as the ventral striatum indicates the smokers did not significantly differ from nonsmokers in terms of reward processing. These results are not surprising considering that monetary gains and losses are secondary reinforcers for both smokers and nonsmokers and indicate that smoking alone does not alter basic reward processing. Moreover, in regions that showed differences between smokers and nonsmokers, those smokers showing the greatest changes in craving also showed the greatest levels of activation. An additional limitation of the current study was the moderate level of nicotine dependence in the current sample with FTND scores ranging from 1 to 8. We predict that the findings regarding sensitivity to punishment would be enhanced at higher levels of dependence due to higher levels of craving and withdrawal.

Overall, these results indicate that sensitivity to punishment may be enhanced following a short period of abstinence and that smokers who are particularly sensitive to punishment tend to crave smoking more to relieve negative effect. These results are particularly relevant to smoking cessation, since increases in craving and negative affect during a quit attempt predict cessation failure (McCarthy et al. 2008; Piper et al. 2010). Future studies should examine whether reward and punishment sensitivity can predict smoking cessation success.
